# Personality and Cognition in Gamers: Avoidance Expectancies Mediate the Relationship Between Maladaptive Personality Traits and Symptoms of Internet-Gaming Disorder

**DOI:** 10.3389/fpsyt.2018.00304

**Published:** 2018-07-10

**Authors:** Christian Laier, Elisa Wegmann, Matthias Brand

**Affiliations:** ^1^General Psychology, Cognition and Center for Behavioral Addiction Research, Universität Duisburg-Essen, Duisburg, Germany; ^2^Erwin L. Hahn Institut für Magnetresonanztomographie, Essen, Germany

**Keywords:** internet-gaming disorder, addiction, personality, use expectancies, internet addiction

## Abstract

Internet-gaming disorder (IGD) has become a clinically relevant phenomenon worth investigating with respect to its mechanisms of development and maintenance. Considering theoretical models of specific Internet-use disorders, we assumed an interaction of maladaptive personality traits as unspecific predisposing factors and experience-based, gaming-related Internet-use expectancies in predicting symptoms of IGD. Therefore, 103 male and female regular Internet gamers were investigated with questionnaires assessing maladaptive personality traits in accordance to DSM-5, gaming-related positive and avoidance Internet-use expectancies, and symptoms of IGD. The results demonstrated that negative affectivity, detachment, antagonism, disinhibition, psychoticism as well as gaming-related positive and avoidance expectancies were related to symptoms of IGD. Moreover, the relationship between maladaptive personality traits as represented by negative affectivity, detachment, and psychoticism with symptoms of IGD was mediated by avoidance expectancies. Positive gaming-related use expectancies were related to detachment, and were not a significant mediator in the hypothesized model. The findings give reason to assume that maladaptive personality traits in combination with gaming-related positive expectancies and avoidance expectancies are important factors for the development of IGD, but that positive expectancies and avoidance expectancies play a differential role regarding there mediating role between personality characteristics and symptoms of IGD.

## Introduction

Most individuals are able to use games in an unproblematic way and do not or only occasionally experience problems related to gaming. Some individuals, however, develop a long-lasting excessive gaming behavior, which is characterized by a repeated loss of control about their use of online games, craving toward gaming, and severe gaming-related negative consequences in everyday life (1, 2). However, the phenomenon's characteristics are still discussed controversially and several researchers from media but also clinical psychology criticize a potential classification of Internet-gaming disorder (IGD) because of its weak scientific basis (3). Also, many researchers share the opinion that it is important not to overpathologize common behaviors in the digital environment of the Internet (e.g., gaming) and to explicitly define exclusion criteria for excessive behaviors that should not be considered pathological (4–6). Other researchers, however, are convinced that IGD and other pathological Internet-related behaviors (e.g., pornography use) should be included in future classification systems of diagnoses and are considered most appropriately as behavioral addictions (7–18). IGD has an estimated prevalence of 0.3–2.5% (16, 19, 20) and has recently been included in the DSM-5 as a condition that warranted further empirical study (21). Inclusion of gaming disorder in the 11th revision of the ICD is currently being proposed (13, 22). Especially against the background of the controversial discussion on the nature of IGD, more research is needed to understand the psychological and neurobiological mechanisms underlying the development and maintenance of IGD symptoms (15, 23).

Theoretical models on psychological processes underlying the development and maintenance of IGD already exist. Such models can inspire theory-driven research to support specific arguments, but also to integrate or disapprove arguments from other psychological models or research fields by testing theory-based hypotheses. By this, theoretical models on IGD can enrich optimization of prevention and treatment methods, if tested empirically and considered valid. The cognitive-behavioral model by Dong and Potenza (24) observes IGD from a decision-making perspective and emphasizes the role of reward sensitivity, executive control, and cognitive biases toward gaming which might enhance or decrease a seeking motivation in terms of craving. Craving is assumed to interfere with decision making in potential gaming-related situations leading to a preference for immediate reward despite potential negative consequences in order to receive positive and/or negative reinforcement by gaming (e.g., reduced stress, increased pleasure). The I-PACE model by Brand et al. (7) focuses on a shift from experienced gratification by gaming to compensation following the repeated use of Internet games and neuropsychological changes on the basis of interactions of personal characteristics, affective, cognitive, and executive processes. More specifically, it is postulated that relationships between predisposing personal characteristics (e.g., personality) with affective and cognitive responses toward addiction-related cues alter during the development of IGD. The tripartite model by Wei et al. (25) explains the addictive process underlying IGD on the basis of a hyperactive impulsive, a hypoactive reflective, and a modulating interoceptive awareness system. While the three models differ in their focus, they have in common that they describe a gradual development of IGD associated with changes in underlying neuropsychological systems for reinforcement and impulse control. Moreover, the cognitive-behavioral model (24) and the I-PACE model (7) hypothesize personality characteristics in form of unspecific predisposing factors involved in the development of IGD. In both models, the effect of personality variables on the development of IGD is considered being indirect and in interaction with other biological, psychological, social, or cognitive factors. Especially the I-PACE model suggests that an interaction of potentially predisposing personality factors with use expectancies and dysfunctional coping styles could be one of several important processes facilitating IGD's development (7). In fact, several studies have already assessed the association between personality traits and IGD symptoms. It was shown that neuroticism, extraversion, openness to experience, agreeableness, and conscientiousness (also known as the Big Five) were associated with IGD (26). Other studies on the relationship of IGD with the five-factor model of personality showed mixed results [cf. (27–29)], but support the finding that the Big Five are related to IGD. Moreover, it was reported that several other personality characteristics such as psychosocial well-being (30), aggression, anxiety, and sensation seeking (31), risk-taking (32), schizotypical traits (33) as well as self-directedness, self-control, and narcissism (34, 35) were related to symptoms of IGD [for review also see (36)]. Furthermore, the DSM-5 includes five maladaptive personality traits which can be broadly understood as maladaptive variants of the five-factor model of personality: Negative affectivity, detachment, antagonism, disinhibition, and psychoticism (37, 38). These factors are described as maladaptive variants of the Big Five and it was reported that the five maladaptive facets of personality are interrelated with moderate effect sizes (39). These personality traits have already been shown to be related to unspecified Internet-use disorder (40), but also other psychological disorders such as depression or alcohol abuse (39, 41).

**Figure 1:**
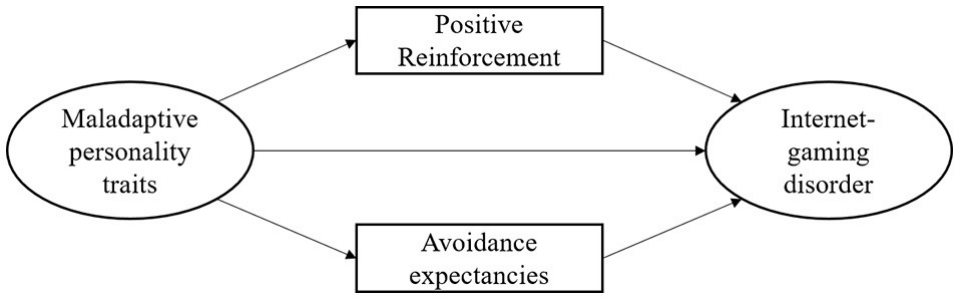
The current structural equation model. Visualization of the main assumptions of the current structural equation model including symptoms of an Internet-gaming disorder as dependent variable.

Further suggestions of the I-PACE model (7) are that gaming-related cognitions (e.g., use expectancies) should be associated with symptoms of IGD and should furthermore mediate the relationship between personal characteristics with symptoms of IGD. Brand et al. (42) were able to show that both positive and avoidance expectancies were associated with symptoms of a generalized/unspecified Internet-use disorder and that they mediated the relationship of psychopathological aspects, personality, and social cognitions with symptoms of an Internet-use disorder. Based on the finding that especially positive expectancies were related to an overuse of diverse Internet applications, such positive expectancies can be considered risk-factors for the development of an Internet-use disorder (43–45) showed that positive use expectancies predicted students' attitudes toward gaming, which in turn predicted symptoms of an Internet-use disorder. However, the relevance of avoidance expectancies as a key mechanism of a dysfunctional Internet use was emphasized in two other former studies, which have investigated potential underlying mechanisms of an Internet-communication disorder (46, 47). The studies illustrated that especially avoidance expectancies increased the risk of a pathological use of social networks since these expectancies comprise the ideas that the use of Internet-communication applications can help to escape from reality, to reduce stress, and to avoid, or reduce negative feelings. Positive expectancies were associated with the experience of pleasure and positive emotions, which seem to be less important in the process of an addictive use of social networks and other Internet-communication applications. Against the background of the I-PACE model (7) and the findings regarding the role of personality and use expectancies in Internet-use disorders, interactions of personality factors and gaming-related use expectancies in predicting symptoms of IGD can be assumed. Since gaming can be considered as behavior with potentially positive and negative reinforcing effects (2, 48), particularly gamers who are prone for the experience of psychological stress or discomfort in everyday life because of maladaptive personality traits should develop gaming-related positive or avoidance expectancies. These expectancies should in turn accelerate the repeated use of online games. In the current study, we aimed at investigating the relationship between symptoms of IGD and the interrelated maladaptive personality traits in accordance to DSM-5 (39) as well as the role of gaming-related use expectancies (7). We hypothesized that (1) symptoms of IGD are related to maladaptive personality traits, such as negative affectivity, detachment, antagonism, disinhibition, and psychoticism, (2) symptoms of IGD are related to gaming-related use expectancies, and (3) gaming-related use expectancies mediate the relationship between maladaptive personality traits and symptoms of IGD. The hypothesized mediation model is demonstrated in Figure 1 below.

## Methods

### Participants and procedure

One hundred and three participants aged between 17 and 59 years (*M* = 28.00, *SD* = 8.27, one gave no information) took part in the current study. Of these, 43 were females and 59 were males, one gave no information. All participants were regular users of online games and played at least once a month. Regarding the usage of online games, we indicated that all questions should be responded by considering the most played and favorite online game. The participants indicated that they played their favorite online game in average 419.97 min per week (*SD* = 470.66, range between 10.00 and 2100.00 min per week). We recruited the student sample at the University of Duisburg-Essen. The study was conducted as online survey and took ~15 min to be answered. The local ethics committee of the University of Duisburg-Essen approved the study.

### Questionnaires

The short-Internet Addiction Test modified for gaming (s-IATgaming, Cronbach's α in our sample = 0.839) was used to assess subjective complaints due to gaming and symptoms of IGD (49, 50). On a scale from 1 (= “never”) to 5 (= “very often”), 12 items (six per factor) of the two factors “loss of control/time management” (s-IATgaming-1, Cronbach's α in our sample = 0.806) and “craving/social problems” (s-IATgaming-2, Cronbach's α in our sample = 0.698) were answered. A sum score was calculated with high tendencies toward IGD being represented by high sum scores. Maladaptive personality traits were measures by the German short-version of the personality inventory for DSM-5 [PID-5-BF, overall Cronbach's α in our sample = 0.798, (51)]. The questionnaire consists of 25 items measuring the personality traits negative affectivity, detachment, antagonism, disinhibition, and psychoticism based on a scale from 0 (= “very false or often false”) to 3 (= “very true or often true”). To assess gaming-related expectancies, the Internet Use Expectancies Scale (IUES) (42) modified for gaming was used. The questionnaire consists of two factors: “positive expectancies” (IUESgaming_positive, Cronbach's α in our sample = 0.847) and “avoidance expectancies” (IUESgaming_avoidance, Cronbach's α in our sample = 0.809), which are represented by overall eight items (four per factor) being answered on a scale from 1 (= “completely disagree”) to 6 (= “completely agree”).

### Statistical analyses

The statistical analyses were carried out using SPSS 25.0 for Windows (52). Pearson's correlation were calculated to analyze bivariate correlations between two manifest variables. To interpret the correlation coefficients, effect sizes were using based on Cohen et al. (53), where *r* ≥ 0.01 indicates a low, *r* ≥ 0.03 indicates a medium, and *r* ≥ 0.05 indicates a high effect. We also investigated the mediation effects in a structural equation model by using Mplus 6 (54). The evaluation of the model fits of the structural equation model is based on the standardized root mean square residual (SRMR; value ≤ 0.08 indicate a good fit with the data), root mean square error of approximation (RMSEA; value ≤ 0.08 indicate a good fit with the data), comparative fit indices (CFI and TLI; values ≥ 0.95 indicate a good fit with the data), as well as on the χ2-Test to control, if the data derivate from the defined model. We also used the method of item parceling for manifest variables, such as the avoidance expectancies, to reduce measurement errors by creating latent dimensions of these variables for the structural equation model (55, 56).

## Results

The mean values and the standard deviations of all questionnaires as well as the bivariate correlations between the variables can be found in Table 1. It could be shown that all variables of the PID-5 as well as the factors of the IUES modified for gaming significantly correlated with the s-IATgaming sum score. However, the Pearson's correlations of the variables indicated that only the negative affectivity, detachment, and psychoticism of the maladaptive personality traits significantly correlated consistently with the IUES modified for gaming, but not the variables antagonism and disinhibition. Additionally, there was no significant correlation with the subscale of the sIATgaming-1 as well as with the PID-5 variables such as negative affectivity and psychoticism. In the next step, we tested the hypothesized structural equation model, which indicated no good fit with the data (SRMR = 0.073, RMSEA = 0.125 and *p* = 0.001, CFI = 0.796, TLI = 0.694, χ2 = 62.53 with *df* = 24 and *p* ≤ 0.001). The results showed that the variables antagonism and disinhibition, which did not correlate with the Internet-use expectancies consistently, did not represent the latent dimension of the maladaptive personality traits very well (β ≤ 0.263). Besides these two variables, we also excluded the variable positive gaming-related expectancies from the structural equation model, which also did not fulfill the requirements of a mediation model.

**Table 1:** Bivariate correlations between the scores of the s-IATgaming and the applied scales.

		***M (SD)***	**2**	**3**	**4**	**5**	**6**	**7**	**8**	**9**	**10**
1. s-IATgaming sum score	IGD	23.20 (6.78)	0.925^**^	0.852^**^	0.234^**^	0.591^**^	0.307^**^	0.352^**^	0.200^*^	0.316^**^	0.495^**^
2. s-IATgaming sum-1		13.11 (4.39)	–	0.589^**^	0.172	0.570^**^	0.245^*^	0.290^**^	0.077	0.336^**^	0.525^**^
3. s-IATgaming sum-2		10.10 (3.19)	–	–	0.260^**^	0.473^**^	0.315^**^	0.349^**^	0.319^**^	0.209^**^	0.330^**^
4. IUESgaming_positive	Internet-use expectancies	4.31 (1.16)	–	–	–	0.262^**^	0.176	0.203^*^	0.171	−0.104	0.080
5. IUESgaming_avoidance		2.48 (1.19)	–	–	–	–	0.404^**^	0.438^**^	0.093	0.158	0.429^**^
6. PID-5 negative affectivity	Maladaptive personality traits	1.25 (0.56)	–	–	–	–	–	0.347^**^	0.098	0.190	0.384^**^
7. PID-5 detachment		0.85 (0.50)	–	–	–	–	–	–	0.165	0.200^*^	0.474^**^
8. PID-5 antagonism		0.50 (0.46)	–	–	–	–	–	–	–	0.320^**^	0.161
9. PID-5 disinhibition		1.04 (0.39)	–	–	–	–	–	–	–	–	0.351^**^
10. PID-5 psychoticism		0.99 (0.63)	–	–	–	–	–	–	–	–	–

* p ≤ 0.050;

** *p ≤ 0.010*.

Therefore, we modified our hypothesized model by integrating only negative affectivity, detachment, and psychoticism as predictors and avoidance expectancies as mediator variable in the structural equation model with IGD symptoms as dependent variable. To represent the “avoidance expectancies” on latent dimensions, we used the method of item parceling, which reduces measurement errors. Therefore, we calculated the bivariate inter-correlations between all items and calculated sub-factors (subfactor 1: *M* = 2.35, *SD* = 1.27; subfactor 2: *M* = 2.60, *SD* = 1.32) for the latent dimension “avoidance expectancies,” which correlated with all used variables consistently (*r*'s ≥ 0.364, *p*'s ≤ 0.001).

**Figure 2:**
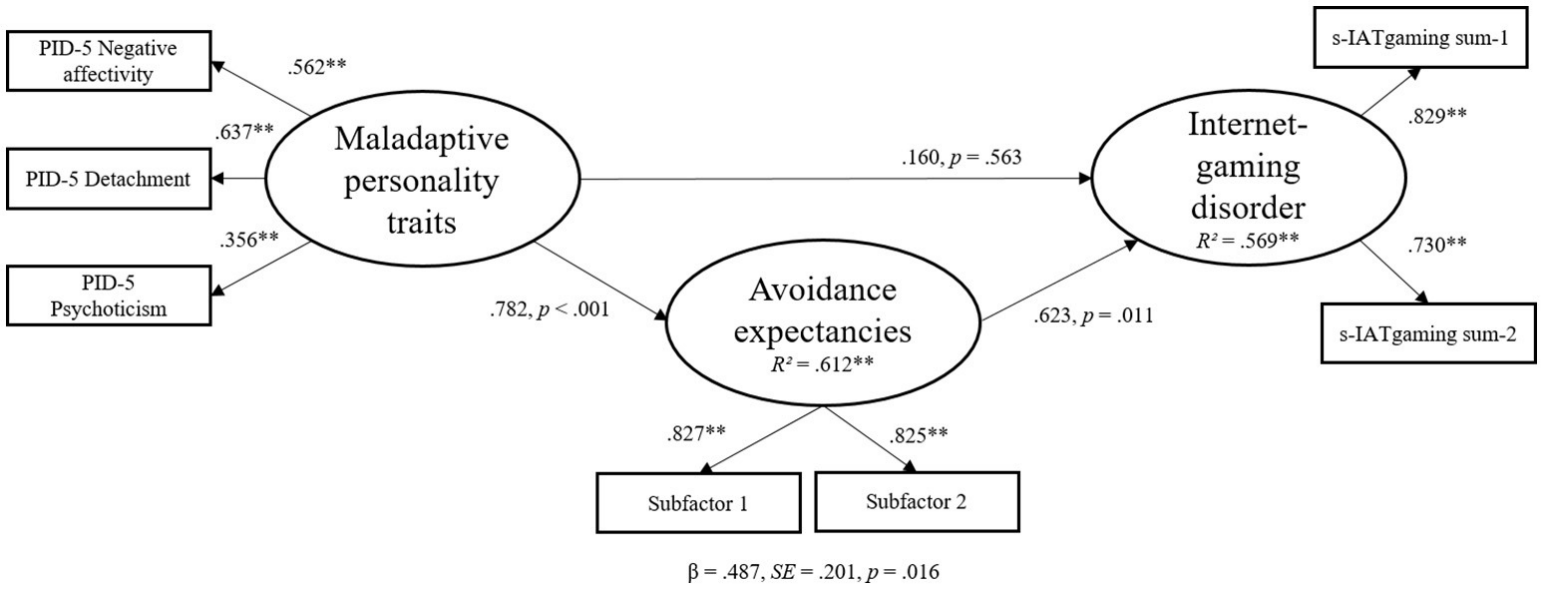
Results of the structural equation model. Results of the structural equation model with IGD as dependent variable including factor loadings on the described latent variables and the accompanying β-weights, *p*–values, and residuals.

The modified version of the structural equation model on latent level is shown in Figure 2. The model showed an excellent fit with the data (SRMR = 0.030, RMSEA ≤ 0.001 and *p* = 0.730, CFI = 1.00, TLI = 1.01, χ2 = 9.89 with *df* = 11 and *p* = 0.541). The defined latent dimensions were well represented by the manifest variables. The results indicate that the latent dimension “maladaptive personality traits” had no direct effect on symptoms of IGD (β = 0.160, *SE* = 0.276, *p* = 0.563), but a significant effect on the latent dimension “avoidance expectancies” (β = 0.782, *SE* = 0.105, *p* < 0.001). Avoidance expectancies also significantly predicted symptoms of an IGD (β = 0.623, *SE* = 0.244, *p* = 0.011). The effect of maladaptive personality traits on symptoms of an IGD was significantly mediated by avoidance expectancies (β = 0.487, *SE* = 0.201, *p* = 0.016) indicating a full mediation effect. Overall, the structural equation model could explain 56.90% of the variance in IGD symptoms.

As an additional analysis, we controlled our results by considering gender as potential variable, which potentially has an impact on the effects. The results showed that female and male participants significantly differed in the personality traits “negative affectivity” and “antagonism,” but there were no significant differences in the main variables of the structural equation modeling measuring IGD or IUES modified for gaming (see Table 2). Considering the small sample sizes, we did not calculated the structural equation model separately for males and females.

**Table 2:** Group comparisons of the s-IATgaming and the applied scales.

	**Female participants (*n* = 43)**	**Male participants (*n* = 59)**	**Group comparison**
1. s-IATgaming sum score	22.58 (6.96)	23.78 (6.65)	*t*_(100)_ = −0.88, *p* = 0.381, *d* = 0.18
2. s-IATgaming sum-1	13.00 (4.38)	13.24 (4.45)	*t*_(100)_ = −0.27, *p* = 0.790, *d* = 0.05
3. s-IATgaming sum-2	9.58 (3.33)	10.54 (3.04)	*t*_(100)_ = −1.52, *p* = 0.133, *d* = 0.30
4. IUESgaming_positive	4.16 (1.03)	4.44 (1.24)	*t*_(100)_ = −1.18, *p* = 0.242, *d* = 0.24
5. IUESgaming_avoidance	2.70 (1.28)	2.33 (1.10)	*t*_(100)_ = 1.53, *p* = 0.128, *d* = 0.31
6. PID-5 negative affectivity	1.48 (0.59)	1.08 (0.49)	*t*_(100)_ = 3.73, *p* ≤ 0.001, *d* = 0.75
7. PID-5 detachment	0.86 (0.48)	0.85 (0.51)	*t*_(100)_ = 0.05, *p* = 0.961, *d* = 0.02
8. PID-5 antagonism	0.39 (0.40)	0.58 (0.49)	*t*_(100)_ = −2.03, *p* = 0.046, *d* = 0.42
9. PID-5 disinhibition	1.01 (0.39)	1.07 (0.40)	*t*_(100)_ = −0.74, *p* = 0.461, *d* = 0.15
10. PID-5 psychoticism	0.99 (0.64)	1.00 (0.62)	*t*_(100)_ = −0.05, *p* = 0.963, *d* = 0.02

## Discussion

The aim of the study was to investigate the relationship between symptoms of IGD with maladaptive personality traits and gaming-related use expectancies. Based on the predictions of the I-PACE model of specific Internet-use disorders (7), we hypothesized that gaming-related use expectancies mediate the relationship between maladaptive personality traits and symptoms of IGD. The main results were that negative affectivity, detachment, antagonism, disinhibition, psychoticism as well as gaming-related positive and avoidance expectancies were related to symptoms of IGD. Moreover, avoidance expectancies mediated the relationship between maladaptive personality traits as represented by negative affectivity, detachment, and psychoticism with symptoms of IGD. The results are generally in line with the I-PACE model and emphasize the differential role of positive and avoidance expectancies in interaction with personality characteristics of the gamers.

Addictive behaviors are considered to develop due to complex interactions between psychological, psychiatric, biological/genetic, sociodemographic, and cultural, factors and within this context premorbid personality traits might increase individuals' vulnerability for developing an addiction (57). This assumption has been included in the I-PACE model of specific Internet-use disorders given that personality factors are mentioned as one potential unspecific factor of vulnerability to develop an addictive use of specific Internet applications (7). It was supposed that personality's contribution to the development of a specific Internet-use disorder is indirect and becomes influential only in interaction with Internet-related cognitive biases (e.g., use expectancies), coping as well as with affective and cognitive responses to the use of a specific Internet applications. Consistent with the results of Gervasi et al. (40) regarding non-specified Internet-use disorder, we found all postulated variables to be related with symptom severity of IGD, we found also that avoidance expectancies mediated the relationship between negative affectivity, detachment, and psychoticism with IGD, while positive expectancies were only related to detachment. Under the consideration of the investigated sample of a mixed sample regarding sex and preferred online games, it needs to be discussed why avoidance expectancies were not related to antagonism and disinhibition and why positive expectancies only correlated with detachment and symptoms of IGD so that consequently the hypothesized model was modified. Overall, the result that all of the maladaptive personality traits were associated with symptoms of IGD was as expected. Since it was postulated that the effect of personality is only indirect and becomes influential only in interaction with Internet-related cognitive biases (7), the findings of the study supports the assumption that especially individuals who are prone to experience psychological discomfort and stress in everyday life because of maladaptive personality. Moreover gamer who have the experience-based expectancy that gaming helps them to avoid these aversive feelings report highest symptom severity of IGD. Avoidance expectancies toward gaming seem to be relevant if individuals report high negative affectivity (i.e., they experience separation insecurity, anxiety, emotional lability, etc.), detachment (i.e., they experience withdrawal, anhedonia, and avoid intimacy), and psychoticism (i.e., they have unusual beliefs and experiences as well as cognitive and perceptual dysregulation). Avoidance expectancies toward gaming seem not be relevant if individuals report high antagonism or disinhibition, since they were associated with symptoms of IGD, but not with avoidance expectancies. Individuals scoring high on antagonism stand out due to manipulativeness, deceitfulness, grandiosity, callousness, and attention seeking, while individuals with high inhibition excel by impulsivity, irresponsibility, risk taking, distractibility, and rigid perfectionism (39). Even if individuals experience aversive mood because of their antagonism or disinhibition (e.g., due to interpersonal problems), the expectancy to avoid aversive mood by gaming seems not to be relevant within this context. The resulting needs from this personality characteristics might neither be fulfilled nor avoided by most online games [which might be potentially relevant when investigating specific game genre like Massively Multiplayer Online Role-Playing Games, see (58)]. Moreover, it could by assumed that avoidance expectancies should not be relevant if someone has a rather risky and impulsive decision making style, because decisions should then not be made deliberately under the consideration of maybe positive or avoidance expectancies. Correspondingly to this argumentation it was shown that impulsivity as a facet of disinhibition is associated with a rather dysfunctional, risky decision-making style in general (59, 60) and that risky decision making is associated with IGD (61). The use of online games under the expectation that the use of games helps to avoid feelings associated with e.g. separation insecurities, anxiousness, emotional lability, intimacy avoidance, or anhedonia could be understood as dysfunctional coping behavior with negatively reinforcing short-term effects with potentially negative effects in the long-run. This conclusion is also consistent with the findings of a study, which showed that the correlation of attachment disorganization amongst players of online role games with symptoms of IGD was mediated by dissociation (62). The authors concluded that dissociation due to gaming might distract gamers from memories of loss, neglect and abuse experienced in the attachment relationships. Another recent study showed that the belief that general Internet use helps to regulate negative emotions and that it affords greater controllability mediates the relationship between deficits in emotion regulation and symptoms of an (unspecified) Internet-use disorder (63). It was also reported that dysfunctional coping is another mediator between symptoms of IGD (64–66) and moreover between symptoms of generalized/unspecified Internet-use disorder, Internet-pornography-use or Internet-communication disorder (67–69). Dysfunctional coping as a result of dysfunctional use expectancies might contribute to the gradual shift from gratification to compensation within the addictive process (7) and consequently be associated with the development of IGD. These findings also support the earlier suggestion to address use expectancies within the cognitive-behavioral treatment of IGD or other Internet-use disorders (42, 70, 71). Considering the earlier findings indicating that use expectancies and dysfunctional coping are associated with IGD (64–66) and that positive expectancies and especially avoidance expectancies seem to be associated with symptoms of several types of specific Internet-use disorders (42, 46, 68, 69), specific expectancies should be addressed in treatment by cognitive restructuring as suggested, for example, by Young and Brand (72).

Positive use expectancies were also related to symptoms of IGD, but were only related to detachment, but not to the other maladaptive personality traits. One may assume that individuals who tend to withdrawal, anhedonia, or intimacy avoidance might have the experience-based cognition that gaming might help them to feel good and experience pleasure and that this might contribute to the development of gaming as a dysfunctional coping mechanism. It could be assumed that gaming-related positive expectancies might also be related to other personality traits such as sensation seeking and that they could be a mediator between these predisposing variables and symptoms of IGD reflecting rather the aspect of gratification postulated to be relevant in the early phases of the development of an addictive behavior (7).

### Limitations and future studies

Since we investigated a non-clinical sample of relatively young male and female adults, who played different online games, the findings need to be interpreted cautiously. The results need to be replicated by investigating a clinical sample of individuals fulfilling the criteria for IGD as suggested by DSM-5 (21). Future studies could investigate adolescents to answer the research question whether the relationship between maladaptive personality, gaming-related use expectancies, and symptoms of IGD is comparable to our study's findings based on the survey of adults. Moreover, the role of meta-cognition should be addressed (73). Generally, it is important to understand whether and how specific types of online games with different reinforcement mechanisms differentially reinforce individuals with different personality types.

## Conclusions

Since IGD is a relatively new phenomenon, the interactions between several predisposing factors and process variables is poorly understood and need to be addressed in more detail. The findings of the current study are one example showing that interactions between different personality traits and use expectancies can explain symptoms of IGD. More studies analyzing interactions between certain variables beyond reporting bivariate effects are needed to contribute to a better understanding of the processes involved in the development and maintenance of IGD and other types of Internet-use disorders.

## Ethics statement

The study was approved by the ethics committee of the division of Computer Science and Applied Cognitive Science at the Faculty of Engineering at the University of Duisburg-Essen. The participants answered the questions in an online-survey which would call for about 30 min of their time. This was the instruction: The participation is voluntary and it is possible to abort at any time without having to state a reason. Your answers will be saved anonymously and treated in confidence, to ensure that no interference to you personally is possible. Personal data cannot be disclosed to third parties and will be only used in the context of this scientific study. By participating you agree that the anonymized results of this study be used for scientific purposes only.

## Author contributions

All authors listed have made a substantial, direct and intellectual contribution to the work, and approved it for publication.

### Conflict of interest statement

The authors declare that the research was conducted in the absence of any commercial or financial relationships that could be construed as a potential conflict of interest.
